# PI3K in T Cell Adhesion and Trafficking

**DOI:** 10.3389/fimmu.2021.708908

**Published:** 2021-08-06

**Authors:** Kristoffer H. Johansen, Dominic P. Golec, Julie H. Thomsen, Pamela L. Schwartzberg, Klaus Okkenhaug

**Affiliations:** ^1^Department of Pathology, University of Cambridge, Cambridge, United Kingdom; ^2^Laboratory of Immune System Biology, NIAID, NIH, Bethesda, MD, United States

**Keywords:** PI3K, integrin, LFA-1, CD62L, CCR7, adhesion, trafficking

## Abstract

PI3K signalling is required for activation, differentiation, and trafficking of T cells. PI3Kδ, the dominant PI3K isoform in T cells, has been extensively characterised using PI3Kδ mutant mouse models and PI3K inhibitors. Furthermore, characterisation of patients with Activated PI3K Delta Syndrome (APDS) and mouse models with hyperactive PI3Kδ have shed light on how increased PI3Kδ activity affects T cell functions. An important function of PI3Kδ is that it acts downstream of TCR stimulation to activate the major T cell integrin, LFA-1, which controls transendothelial migration of T cells as well as their interaction with antigen-presenting cells. PI3Kδ also suppresses the cell surface expression of CD62L and CCR7 which controls the migration of T cells across high endothelial venules in the lymph nodes and S1PR1 which controls lymph node egress. Therefore, PI3Kδ can control both entry and exit of T cells from lymph nodes as well as the recruitment to and retention of T cells within inflamed tissues. This review will focus on the regulation of adhesion receptors by PI3Kδ and how this contributes to T cell trafficking and localisation. These findings are relevant for our understanding of how PI3Kδ inhibitors may affect T cell redistribution and function.

## Introduction

PI3K signalling controls numerous pathways that are involved in regulating trafficking and localisation of T cells between lymphoid system and organs, and tissues through the circulatory and lymphatic systems. During the process of T cell migration, integrins are crucial mediators of adhesion and are extended to an open high-affinity conformation following stimulation of chemokine receptors and/or T cell receptor stimulation. The major integrin expressed on T cells is Leukocyte Function-associated Antigen 1 (LFA-1), which is expressed on all subsets of T cells as well as other leukocytes, including B cells and neutrophils. LFA-1 mediates T cell transendothelial migration as well as formation of a stable immunological synapse with antigen presenting cells (APC). The mechanistic regulation of LFA-1 affinity has been extensively studied since its discovery in 1981 as a target for monoclonal antibodies inhibiting cytotoxic T cell-mediated killing (1–3), yet many questions remain about its precise regulation and function. Besides LFA-1, several other adhesion molecules are involved in T cell migration, including L-selectin (CD62L) found on naïve T cell subsets and on central memory T (T_CM_) cells. CD62L binds ligands such as GlyCAM-1 and CD34 expressed on endothelial cells and is required for efficient naïve T cell homing to LNs through high endothelial venules (HEV) [reviewed in (4)].

In this article we will review how PI3K signalling regulates T cell adhesion, migration and localisation by regulating CD62L and LFA-1 affinity, as well as how this can be targeted by PI3K inhibition. Cytokines and chemokines are also essential for coordinating the trafficking of lymphocytes. Of these, the expression of CCR7 and IL7Rα (CD127) are negatively controlled by PI3Kδ signalling in a FOXO1-dependent manner and will also be considered.

## PI3K Signalling in T Cells

Class I PI3Ks phosphorylate the D3-position of the inositol ring of PtdIns (4,5)P_2_ (PIP_2_) to generate PtdIns (3,4,5) P_3_ (PIP_3_). PIP_3_ is bound by a subset of pleckstrin homology (PH) and other PIP_3_-binding domains. Proteins with PIP_3_-binding properties are hence recruited to the membrane resulting in initiation of downstream signal transduction. The class I PI3K subfamily is comprised of class IA PI3Ks (PI3Kα, PI3Kβ, and PI3Kδ) and class the IB PI3K (PI3Kγ). The class I PI3Ks are heterodimeric proteins consisting of a regulatory domain (class IA PI3Ks; p85, class IB PI3K; p101) and a catalytic domain [p110α (PI3Kα), p110β (PI3Kβ), or p110δ (PI3Kδ), or p110γ (PI3Kγ)] (5). Class II and class III PI3Ks use PtdIns or PtdIns (4)P as a substrate and are involved in intracellular membrane trafficking, these will not be considered here [reviewed in (6)].

**Figure 1:**
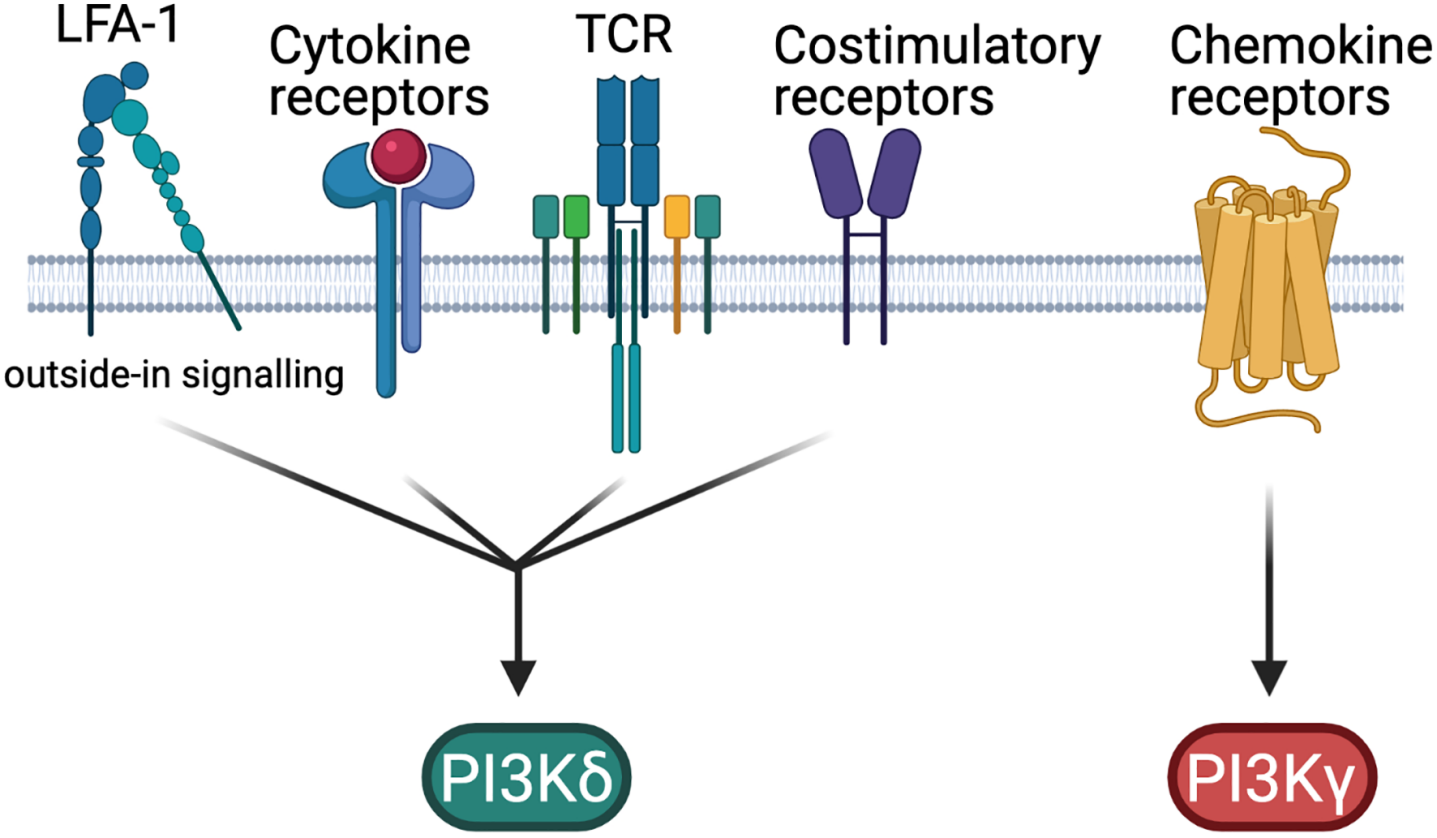
PI3K activation in T cells. Simplified schematic of the differential regulation of PI3Kδ and PI3Kγ in T cells. Figure made in BioRender.

In T cells PI3Kδ is the dominant class I PI3K isoform. PI3Kδ is activated downstream of the TCR as well by costimulatory and cytokine receptors, that stimulate the phosphorylation of tyrosines within YXXM motifs that bind to the SH2 domains of the p85 subunit (7) (**Figure 1**). Indeed, LFA-1 can also activate PI3Kδ *via* so called outside-in signalling (8). PI3Kγ is also expressed in T cells and predominantly mediates signals downstream of G protein-coupled receptors such as chemokine receptors (5) (**Figure 1**).

### PI3K in T Cell Development

Signalling through class I PI3K plays important roles at multiple stages of T cell development. Loss of both p110δ and p110γ results in near complete ablation of thymocyte β-selection, while individual loss of p110δ and p110γ individually only causes minor perturbations of T cell development (9–11). These findings revealed an unexpected redundancy between p110δ and p110γ in developing T cells and was explained by cooperative signalling from the chemokine receptor CXCR4 *via* p110γ and pre-TCR signalling *via* p110δ, either of which is sufficient to generate PIP_3_ required during thymocyte β-selection (12). Consistent with this, deleting the PIP_3_ phosphatase PTEN bypasses the requirement for pre-TCR stimulation during thymic β-selection, presumably by enabling sustained CXCR4-dependent PIP_3_ levels (13). Beyond thymocyte β-selection, mice lacking PTEN show impaired thymocyte negative selection and evidence of autoimmunity, suggesting a role for PI3K activity in maintaining central tolerance (14). In addition, mice expressing a kinase-dead p110δ show increased numbers of Treg within the thymus, highlighting a role for p110δ in thymic T_reg_ selection (15). Together these findings underpin an important role for PI3K signalling in the development of T cells.

Following thymic development, class I PI3K signalling is also crucial for the peripheral development of multiple subsets of T cells; T follicular helper cells (T_FH_ cells) require ICOS signalling during development, and ICOS mutant mice that do not activate downstream PI3Kδ, as well as mice lacking p110δ in T cells, lack T_FH_ cells (16, 17). The loss of T_FH_ cells is the main reason underpinning the lack of germinal centres and immunoglobulin class switching after immunisation of PI3Kδ-deficient mice (16). PI3Kδ also regulates the differentiation of other T_H_ subsets, including T_H_1, T_H_2, T_H_17, and T_reg_, as well as production of cytokines and granzymes in both CD4^+^ and CD8^+^ T cells (15, 18–26). Human patients with loss of function mutations in PI3Kδ have also been identified (27–30). These have a more profound defect in B cell development than observed in mice. By contrast, profound defects in T cell development have not been observed so far in patients lacking the p85α or p110δ subunits of PI3Kδ; but this has been difficult to evaluate systematically in such immune-deficient patients who also suffer from inflammatory disease.

### Activated PI3Kδ Syndrome

More recently, gain-of-function mutations that lead to increased PI3Kδ activity have also shown to be the cause of a novel immunodeficiency syndrome called Activated PI3K delta Syndrome (APDS) (31–35). APDS patients show increased susceptibility to airway infections (e.g. with *Streptococcus Pneumoniae*), chronic virus infections (CMV and EBV) and pertinent to this review, have enlarged LNs and spleens as well as signs of autoimmunity, mainly manifested as cytopenia (34). Remarkably after a 12-week trial of the PI3Kδ inhibitor Leniolisib, the LNs and spleens of these patients reduced in size by up to 50% (36). This may reflect in part the potential of PI3Kδ inhibitors to cause redistribution of lymphocytes in addition to the inhibitory effect on lymphocyte proliferation. Several groups have generated mouse models of APDS which recapitulate many of the features of the patients, including increased susceptibility to airway infections, enlarged LNs and spleen and production of autoantibodies (37–41). Altogether, these studies shine light on the paradox that both loss-of-function and gain-of-function of PI3Kδ leads to immunodeficiency, and highlight how this pathway needs to be dynamically regulated for optimal lymphocyte development and function (42, 43). This, as we will see, is also key for the control of lymphocyte trafficking.

Currently four different PI3Kδ inhibitors are approved for the treatment of B cell malignancies (44). A detailed description of these is beyond the remit of this review, however two concepts learned from the treatment of these patients are worth noting. Chronic lymphocytic leukaemia (CLL) patients treated with PI3Kδ inhibitors such as Idelalisib initially experience a dramatic redistribution of the malignant B cells from the LNs (45). This phenomenon is referred to as lymphocytosis and is now recognised as a beneficial clinical feature of this class of drugs. Lymphocytosis is thought to be secondary to the interference with BCR-dependent integrin activation and chemokine responsiveness (46). CLL cells that are purged from their protective LN environment are more susceptible to undergo apoptotic cell death which can be accelerated with chemotherapy or drugs such as rituximab (anti-CD20) (45). Immune-mediated colitis and hepatitis are common adverse effects of PI3Kδ inhibitors, but skin inflammation is also seen in some studies (47). These are thought to be caused by the selective depletion or inactivation of Tregs, especially from tissues with high exposure to microbial antigens, such as the gut, liver and skin (44, 48). In this context, by targeting Treg, PI3Kδ inhibitors can unleash potent antitumour immune responses (49). Recent evidence suggests that PI3Kδ inhibitors can purge Treg from the tumour microenvironment and into the circulation (50). Hence the capacity of PI3Kδ inhibitors to not only affect lymphocyte function, but also to cause redistribution out of lymphoid tissues may underpin the therapeutic effects of PI3Kδ inhibitors.

## Integrins in T Cell Localisation, Migration, and Adhesion

Integrins are transmembrane, heterodimeric proteins that are involved in cell-cell and cell-extracellular matrix interactions as well as binding of soluble ligands. In mammals the heterodimeric transmembrane structure of integrins is composed of one of 18 α subunits and one of eight β subunits, that can form up 24 combinations. Integrins are involved in T cell migration and localisation within tissues, where conformational priming (activation) of the integrins by intracellular signalling events (“Inside-out” signalling) results in high affinity binding of their ligands. Further, integrins mediate signal transduction, where binding of their ligands stimulates intracellular signalling pathways (“outside-in” signalling).

**Figure 2:**
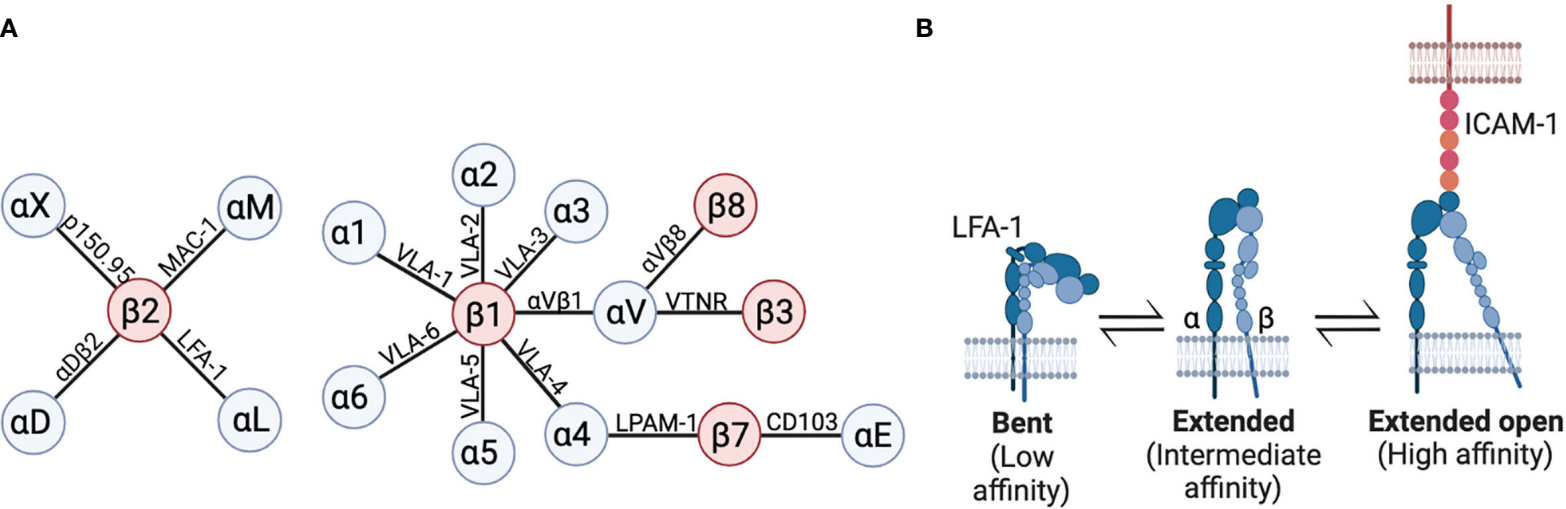
Integrins in T cells. **(A)** Schematic of integrin chains expressed in T cells with α integrin chains in blue, and β integrin chains in red. Lines indicate which integrin chains form heterodimeric integrins, and names over lines are commonly used names of the resulting integrin. **(B)** LFA-1 (αLβ2) integrin in a bent/closed conformation with low affinity, extended/closed conformation with intermediate affinity, and extended open conformation with high affinity. Figure made in BioRender.

T cells are known to express at least 15 of the 24 known integrins depending on their differentiation and activation state (51, 52) (**Figure 2A**). LFA-1 (αLβ2) is expressed by all T cell subsets and specifically binds Intercellular Adhesion Molecules (ICAMs) and Junctional Adhesion Molecules (JAMs) (53, 54). Under steady state, LFA-1 is found in a closed conformation which has low affinity for its ligands. However, following inside-out mediated activation by chemokines, cytokines, or TCR-stimulation, LFA-1 rapidly changes conformation from its low affinity closed/bent conformation to an intermediate affinity extended conformation, where the extracellular domain is partly open, but the cytosolic domain remains closed. This intermediate affinity extended conformation allows for binding to ICAM-1, which can further increase affinity through outside-in signalling resulting in the high affinity open-extended conformation (**Figure 2B**) reviewed in (51, 55). Multiple other integrins are expressed in subsets of T cells, including Very Late Antigen 4 (VLA-4) (α4β1) which binds VCAM-1, however in this review we will focus on the roles of LFA-1.

### Integrins in T Cell Migration

T cells recirculate between LNs through the blood, probing antigen-presenting cells for their cognate antigen. To exit blood vessels, selectins, integrins, and chemokines are required to halt the T cells at the right place and resist the shear stress in the blood (1-70 dyn/cm^2^) (56). This is a tightly regulated process. Selectin-mediated binding of their ligands facilitates rolling along the endothelial membrane which slows down the T cells. This allows the T cells to respond to chemokines secreted from the endothelia and immobilised on glycosaminoglycans (GAGs) on the surface of the endothelial cells Reviewed in (57). As a result, integrins (such as LFA-1 and VLA-4) are activated. LFA-1-mediated binding of endothelial ICAMs (ICAM-1 and -2) leads to firm adhesion to the endothelial barrier. This allows the T cells to crawl against the flow towards chemotactic gradients until the cell will undergo transendothelial migration (TEM, also termed diapedesis) through the endothelial barrier into the underlying tissue.

Recirculation and homing of naïve T cells to secondary lymphoid organs (SLOs), including LNs, requires expression of the chemokine receptor CCR7 and CD62L, both which are downregulated following PI3K activation as discussed later. CD62L interacts with peripheral node addressins (PNAds) expressed on high endothelial venules (HEVs) which are formed by specialised endothelial cells lining post-capillary venules associated with lymph nodes (LNs). The interaction between CD62L and PNAd causes T cells to start rolling along the HEVs. After slowing down, CCR7 on the T cells binds CCL21 presented by HEVs (58, 59), which rapidly induces LFA-1 activation, leading to arrest and transendothelial migration (60, 61). The process of LN entry is highly dependent on LFA1; LFA-1-deficient mice have greatly reduced adhesion to HEVs, particularly in peripheral LNs (pLNs) and therefore elicit limited to no migration to the LNs (62, 63). Similarly, LFA-1-blocking antibodies block adhesion to HEVs and prevent repopulation of LNs (64). Together, HEVs thus function as a selective gateway to the LNs, attracting naïve and resting memory T cells, but largely blocking entry of other leukocytes such as neutrophils under steady state (65). Migration to gut-associated lymphoid tissues, spleen and inflamed lymphoid tissues are governed by other mechanisms and molecules, such as α4β7/MAdCAM-1 interactions, and this integrin seems to be regulated by different pathways than LFA-1 and VLA-4 (66). After entering LNs, the role of LFA-1 is less clear; studies of LFA-1-deficient T cells indicate that LFA-1 is required for retention of T cells in the parenchyma (67). However, other studies using LFA-1-deficient T cells (68) or dendritic cells lacking integrins altogether (69), suggest that interstitial and intranodal motility of T cells and DCs in the absence of antigen is much less dependent on integrins than is the entry into and egress out of the LNs.

Following screening of antigen within the LNs, T cells will egress through the efferent lymphatics in a process regulated by LFA-1/ICAM-1-interactions (67). Egress is guided by the lipid sphingosine-1-phosphate (S1P). S1P is found in low concentrations inside the LNs, but high concentrations in lymph and blood, creating a gradient which attracts the T cells through binding of the GPCR S1P receptor 1 (S1PR1) expressed by naïve T cells (70). Following egress through efferent lymphatics, the lymphatics connect LNs in series, but eventually merge with the thoracic duct allowing the T cells to recirculate through the blood. Interestingly, S1P-mediated egress from inflamed tissues is also partially dependent on interactions between LFA-1/ICAM-1 and VLA-4/VCAM-1. Here LFA-1/ICAM-1 are required for effective migration of memory T cells through afferent lymphatics into LNs (71–73).

Antigen-stimulated T cells downregulate L-selectin and glycosylate P-selectin glycoprotein ligand-1 (PSGL-1) resulting in functional PSGL-1 ligand which allows for binding of L-, P-, and E-selectins that are upregulated on inflamed endothelial tissues reviewed in (4). Other integrins are also involved in migration along, and adhesion to vascular endothelium as well as transendothelial migration of antigen-stimulated T cells. Of particular importance are VLA-4/VCAM-1, α4β7/MAdCAM-1, and αV integrin-mediated (74) interactions that facilitate migration to distinct inflammatory sites reviewed in (75, 76).

### Integrins in the Immunological Synapse

Within the T cell follicles of the LNs, T cells recognise their cognate antigen-MHC complex on the surface of antigen presenting cells (APCs) or target cells. This induces TCR signalling and triggers the formation of an immunological synapse (IS) at the contact area between the T cell and the APC/target cell (77). The IS is a highly specialised and dynamic cell-cell interface that allows for fine-tuning of signalling events leading to T cell activation (78, 79). Integrins and especially LFA-1 are key components in IS formation. In the immature IS, PI3K-dependent chemokine-mediated LFA-1 activation initiates the adhesive contact between T cells and APCs/target cells allowing the T cell to scan its interaction partner for cognate antigens (80). Concomitantly, LFA-1 triggers recruitment of organelles such as mitochondria to the IS thereby preparing the T cell for optimal TCR-induced activation and Ca^2+^ signalling during later activation stages (80).

**Figure 3:**
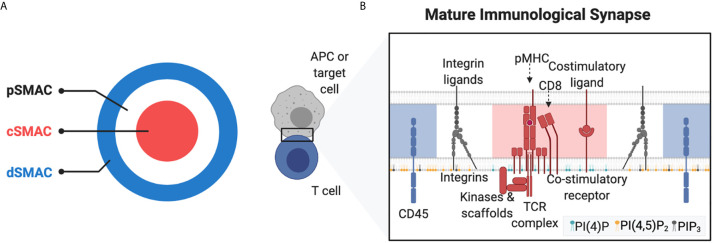
Phospholipids in the immunological synapse. **(A)** shows the bulls eye of the synapse in the Z-plane. **(B)** Schematic of the immunological synapse showing approximate location of critical receptors in the distal SMAC (dSMAC), peripheral SMAC (pSMAC), and central SMAC (cSMAC) as well as approximate composition of the phosphoinositols PI (4)P, PI (4,5)P_2_ and PI (3,4,5)P_3_ in the inner leaflet of the T cell. Figure made in BioRender.

In the immature IS, LFA-1 is found at the centre of the synapse with TCRs and downstream kinases clustered at the periphery (81, 82). During maturation of the IS, the synapse is reorganised into annular supramolecular clusters (SMACs) allowing for spatiotemporal clustering of receptors, adhesion molecules, and signalling effector proteins (**Figure 3A**) (83). In the mature IS, centripetal movement relocates TCR/pMHC complexes to the central SMAC (cSMAC) together with their co-stimulatory molecules, intracellular kinases, and adaptor proteins. Simultaneously, LFA-1/ICAM-1 complexes are redistributed to the integrin-enriched peripheral SMAC (pSMAC) surrounding the cSMAC (81). In the pSMAC, LFA-1 both stimulates T cell activation by increasing the accumulation of TCR/pMHC complexes in the cSMAC, and recruiting signalling molecules to the pSMAC; LFA-1 may also help segregate the phosphatase CD45 to the distal SMAC (dSMAC) (84, 85). In addition to LFA-1, the α4β1 integrin, VLA-4, is also enriched in the pSMAC and is involved in T cell activation by regulating the mobility of SLP-76, an essential adaptor protein functioning downstream the TCR (86). It is thought that VLA-4 can restrain SLP-76 in the pSMAC, so that SLP-76 both remains in closer contact with its upstream activators and avoids the cSMAC, where signalling complexes will eventually be internalised and degraded to terminate TCR signalling (86, 87). Collectively, an important function of integrins in the IS appears to be regulating the localisation of both inhibitory and stimulatory signalling molecules. The phosphatidylinositol (PIP) composition of the IS might also contribute to this spatial regulation of signalling proteins within the IS (**Figure 3B**). Early studies confirmed the accumulation of PIP_3_ inside and outside the IS between APCs and T cells (88–90). However, PIP_3_ seemed less concentrated at the cSMAC, and later studies using transgenic CD8^+^ T cells have found that PI3K-generated PIP_3_ seems specifically accumulated in the periphery of the IS (91–93). Further, both PIP_2_ and PIP_3_ are cleared from the cSMAC during conjugate formation, and sustained PI3K activity is necessary for proper T cell activation possibly through the regulation of PIP_3_ binding proteins (91–93). It has further been suggested that PI3K-dependent actin remodelling in the periphery of the IS can mediate synaptic force on the target cell thereby potentiating target cell killing by CD8^+^ T cells (94).

### Integrin Affinity Regulation in T Cells

Inside-out signalling in T cells is a complex process mediated by a range of proteins that collectively result in increased LFA-1 affinity and avidity. Following TCR stimulation, multiple proteins are recruited to phosphorylated CD3 ITAMs, including the tyrosine kinase ZAP-70. ZAP-70 consequently phosphorylates tyrosine residues of the scaffolding protein LAT. These residues act as anchors for a range of T cell signalling proteins, including SLP76, which is also phosphorylated by ZAP-70. The pSLP76/LAT-complex functions as a scaffold for downstream effector proteins, including PLCγ (95). PLCs catalyse the hydrolysis of PIP_2_ to generate the second messenger signalling molecules diacylglycerol (DAG) and inositol 3-phosphate (IP_3_). In turn, IP_3_ stimulates the release of Ca^2+^ from the endoplasmic reticulum (ER). Ca^2+^ and DAG stimulate activation of the RAP1 guanine exchange factor (GEF), CalDAG-GEFI (also known as RASGRP2) which activates RAP proteins by exchanging GDP for GTP (96). In T cells, RAP1 is a dominant isoform, with both Rap1a and Rap1b being expressed. Of note however, CalDAG-GEFI is not expressed in mouse lymphocytes, suggesting other RAP GEFs are involved in the regulation. Another pathway leading to RAP1 activation is recruitment of the CRKL-C3G complex by the WAVE2-Arp2/3-Abl complex (97, 98). This results in activation of the RAP-GEF, C3G (Also known as RAPGEFI), thus further activating RAP1. Active GTP-bound RAP1 is critical for the process of LFA-1 activation (99–103).

Chemokine receptors are GPCRs that following chemokine binding induce a multitude of signals, some which converge in activation of the small GTPase RAP1 *via* the activation of phospholipase Cβ (PLCβ) which also hydrolyses PIP_2_ to DAG and IP_3_ (104–106). Besides activating the PLC-dependent signalling-cascade, chemokine receptors also induce activation of PI3Kγ resulting in initiation of PI3K-mediated signals discussed further below.

GTP-bound RAP1 interacts with RIAM (107–109) and RAPL (100). In turn, this complex mediates activation (109), and plasma-membrane binding of TALIN1 (108). The FERM3 (F3) domain of TALIN1 in turn binds the β chain of LFA-1 thereby mediating conformational activation of LFA-1 from low to intermediate affinity, as well as mediating downstream cytoskeletal remodelling (110, 111). RIAM has a PH domain that preferentially interacts with PIP_2_. By binding PIP_2_ RIAM is thought to act as a proximity detector mediating binding of activated RAP1 and TALIN1 to the membrane (112). Another PH-domain containing protein involved in the process is SKAP1 (also known as SKAP55). SKAP1 is constitutively associated with ADAP (also known as FYB) and has been shown to also mediate binding of RAP1 to the plasma membrane through its PH domain (113, 114). Together SKAP1/ADAP integrates with the RIAM-RAPL-RAP1 complex during TCR-induced LFA-1 activation, and likely stabilises the complex (115). In parallel during LFA-1 activation, KINDLIN-3 binds the cytoplasmic tail of β integrins and is required for stabilisation of the high affinity conformation of LFA-1 (116–118). TALIN1 thus mediates conformational maturation to an intermediate affinity of LFA-1, whereas binding of both KINDLIN-3 and TALIN1 to the β-chain results in the high affinity conformation of LFA-1 (116).

Several negative regulators of LFA-1 activation exist. RhoH is required to keep LFA-1 in a non-adhesive state (119). RhoH also contributes to TCR signalling by interacting with ZAP70 and LCK (120, 121). Interestingly, chemokine-induced LFA1 activation is suppressed by RhoH, whereas RhoH enhances TCR-induced LFA-1 activation, suggesting that RhoH can divert T cells from chemotactic towards antigen-dependent response (122). CBL-B is an E3 is ubiquitinase that can suppress LFA-1 activation by interfering with the capacity of CRK-L to recruit and activate C3G (123).

Together these intricate regulatory mechanisms integrate migratory signals, such as chemokines, and TCR engagement with integrin activation. Consequently, LFA-1 affinity is turned on and off in a highly regulated manner by multiple microenvironmental cues.

## PI3K-Mediated Activation of Integrins – a Gatekeeper of Antigen-Dependent Adhesion

Box 1 **Affinity Contra Avidity of Integrins – Note of Caution.**

When studying integrins such as LFA-1, regulation can either be modulated by direct changes to affinity by inside-out signalling and outside-in signalling, changes in avidity through surface clustering of the integrins, and lastly by levels of expression or presence at the surface. Studies investigating LFA-1-mediated adhesion often do not clearly distinguish these mechanisms and it is often unclear whether particular mechanisms affect LFA-1 affinity or avidity.

In human T cells antibodies specific to the intermediate or high affinity conformation of LFA-1 can be used to measure affinity, however similar affinity-specific antibodies are not as well established for mouse T cells. Binding of ICAM-1 can be used as a proxy for LFA-1 activity and here binding to single ICAM-1 molecules is more dependent on affinity changes and LFA-1 surface expression, whereas binding of ICAM-1-coated surfaces (immobilised ICAM-1) or conjugate-formation is dependent both on changes to overall avidity as a result of increased affinity, surface expression, and clustering.

Early studies of PI3Ks roles in CD4^+^ T cell activation found that broad inhibition of PI3Ks with Wortmannin reduced antigen-specific interactions between DO11.10 CD4^+^ T cells and OVA-pulsed B cells, as well as T cell adhesion to immobilised ICAM-1 (ICAM-1-coated plastic) (124, 125). Further, Wortmannin was found to inhibit CD28-induced activation (126), and Wortmannin and LY294002 (class I PI3K inhibitor) inhibit CD7-induced activation of β1-integrin-mediated adhesion (VLA1-6) to immobilised fibronectin (127). In accordance, overexpression of a hyperactive p110-CAAX mutant increased ICAM-1-binding in response to PDBu/Ionomycin (95). These early findings all supported a role for PI3K in activation of integrins downstream of TCR-engagement, although caution must be taken with some of these inhibitor studies, as that Wortmannin can affect multiple kinases. Further, caution must be taken when evaluating affinity vs avidity in these studies (**Box 1**).

Further supporting a role for PI3K in integrin activation, kinase-dead p110δ^D910A^ CD4^+^ T cells had reduced affinity towards ICAM-1 after stimulation with anti-CD3, as measured by binding of soluble recombinant ICAM-1 by flow cytometry. As a consequence, OT-II transgenic p110δ^D910A^ CD4^+^ T cells did not form conjugates with OVA_323-339_-pulsed B cells as well as WT OT-II T cells. p110δ^D910A^ mutant T cells had reduced RAP1-GTP, indicating a role for PI3Kδ in RAP1-GTP activation. Interestingly, the activation of LFA-1 was less dependent on AKT suggesting other PIP_3_-binding proteins were responsible for the PI3K-mediated activation of LFA-1 (128).

Treatment of lymphocytes with Wortmannin or LY294002 decreases SDF1α, CCL19, and CCL21-mediated adhesion to ICAM-1. However, this decrease seemed to rather be a consequence of decreased avidity than affinity as a result of decreased chemokine-induced LFA-1 mobility following PI3K inhibition (60). Indeed, chemokine-dependent migration of T cells was largely PI3Kγ-independent and instead mediated by DOCK2 (129). Similarly, interstitial migration and S1P-mediated egress was independent of PI3Kγ (130). PI3Kδ signalling was also not required for chemokine-induced LFA-1 activation (128). Therefore, PI3Kδ activity downstream of TCR-stimulation increases LFA-1 affinity, whereas PI3Kγ-signalling seems dispensable for chemokine-induced LFA-1 affinity regulation.

### Regulation of LFA-1 by PH-Domain Containing Proteins

**Figure 4:**
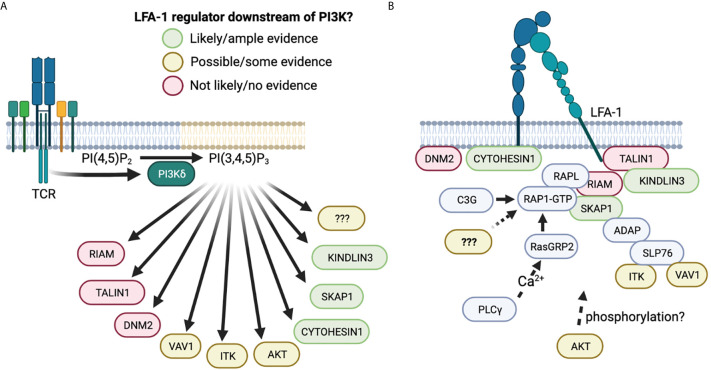
LFA-1 regulators downstream of PI3Kδ. **(A)** Schematic of proteins that have been implicated in LFA-1 regulation, and the likelihood that they are regulated by PI3K based on the literature. Green proteins have ample evidence that they are regulated by PI3K and have been implicated in LFA-1 regulation in multiple studies. Orange proteins have some evidence suggesting that PI3K regulates their functions and are to some extent involved in LFA-1 regulation. Red proteins have PH domains, but are unlikely to be regulated by PI3K due to low PIP_3_ affinity. It is important to note that PIP_2_ is up to 100X more abundant on the plasma membrane than PIP_3_ (131). Therefore, a given protein needs to have high selectivity for PIP_3_ over PIP_2_ in order to be directly regulated by PI3Ks. **(B)** Simplified schematic of how the proteins in **(A)** are involved in regulation of LFA-1, showing interaction partners and approximate location. Figure made in BioRender.

Multiple proteins involved in the process of LFA-1 activation, including CYTOHESIN-1, SKAP1, and KINDLIN-3, have PH domains that bind PIP_3_ and may hence regulate LFA-1 affinity in a PI3K dependent manner (**Figures 4A, B**).

#### CYTOHESINs

The intracellular ARF-GEF protein, CYTOHESIN-1, was described early on to bind β2 integrins (e.g. LFA-1, MAC-1) and activate LFA-1-mediated adhesion to immobilised ICAM-1. The PI3K-mediated membrane recruitment of the PH domain of CYTOHESIN-1 was found to partially facilitate the CYTOHESIN-1-mediated activation of LFA-1 (132–134). CYTOHESIN-1 binds directly to the cytoplasmic tail of β2 integrin, and this interaction as well as the ARF-GEF functionality of its SEC7 homology domain have been shown to regulate the activation of LFA-1 in T cells and LFA-1 mediated transendothelial migration (135, 136). Moreover, CYTOHESIN-1 further regulates activation of RhoA and integrin activation in dendritic cells (137). Surprisingly, CYTOHESIN-1 seems to have opposing roles in regulation of MAC-1 (αMβ2) integrin-mediated adhesion to fibrinogen by neutrophils, suggesting a more complex involvement of CYTOHESIN-1 in regulation of integrin activation (138). This potentially hints a differential role of PI3K-signaling in regulating integrins in different immune subsets depending on their integrin expression. Other CYTOHESIN molecules have also been implicated in integrin regulation, but rather seem to rather be involved in the recycling of integrins from the surface. Whereas CYTOHESIN-2 (ARNO) seems to increase β1 integrin-mediated adhesion and recycling, CYTOHESIN-3 (GRP1) results in decreased adhesion (139), and these opposing effects of CYTOHESIN-2 and -3 were dependent on phosphoinositide specificity (140). How the CYTOHESINs divergently regulate integrins, and further, the mechanism by which PI3K regulates CYTOHESIN-1-mediated LFA-1 activity is still unclear, but it is likely due to dominant negative effects between the different homologs, and should highlight the importance of not treating all integrin signalling pathways equally.

#### SKAP1

SKAP1 is recruited to SLP-76 *via* the adaptor protein ADAP. ADAP/SKAP1 then binds the Rap1-interacting protein, RIAM and contributes to TCR-induced inside-out LFA-1 activation and clustering by supporting formation of RAP1/RAPL complexes as well as membrane recruitment of these essential proteins involved in LFA-1 activation (113, 115, 141, 142). The SKAP1 PH domain was found to be required for membrane recruitment, and this in turn was necessary for the recruitment of RAPL to the membrane (114). A SKAP1 mutant that was constitutively associated with the membrane by addition of a myristoylation site disrupted the requirement for PI3K signalling in binding immobilised ICAM-1 suggesting that PI3K-mediated activation of LFA-1 is dependent on ADAP/SKAP1/RIAM signalling. Indeed, although RIAM contains a PH domain with high affinity for PtdIns monophosphates *in vitro*, SKAP1 is required for recruitment of Rap1/RIAM to the membrane during LFA-1 activation (142). In accordance, K152E mutation of SKAP1 eliminated PIP_3_-binding *in vitro* and as a result impaired immobilised ICAM-1-binding (143). Unexpectedly, this mutant did not abolish SKAP1/ADAP/RIAM/RAPL binding to the membrane, suggesting redundancy in the pathways resulting in membrane-recruitment of these proteins. Surprisingly, SKAP1 mutants lacking the PH domain do not significantly alter its role in integrin-mediated adhesion, suggesting the mechanism by which PI3K regulates SKAP1 is still incompletely understood (113, 143, 144).

#### KINDLIN3

Mutations of the crucial LFA-1 regulator, KINDLIN-3, are the cause of leukocyte adhesion deficiency III (LAD-III), a rare autosomal disorder, resulting in severe bleeding and life-threatening infections as a result of defective β1- and β2-integrin-mediated adhesion (145, 146). Studies by Hart et al. suggest that KINDLIN-3 has higher affinity to PIP_3_ than PIP_2_ (147). They also found that the PIP_3_-binding was necessary for the function of KINDLIN-3, as KINDLIN-3 mutants that specifically did not bind PIP_3_ failed to rescue adhesion of LAD-III cells to ICAM-1. KINDLIN-3 was also found in structural studies to bind PIP^3^ with higher affinity than PIP_2_ (148) and the PH domain was found to regulate the translocation of KINDLIN-3 to the surface membrane in neutrophils (149). These studies therefore suggests that KINDLIN-3 is at least partially regulated by PI3K, though this has yet to be confirmed in lymphocytes.

Thus, several studies indicate a key role for PI3K effector molecules in regulating integrin affinity/avidity, and multiple other proteins have been implicated in PI3K mediated integrin regulation indirectly (**Figure 4B**).

#### Regulation of the RHO Family of GTPases

The RHO family of GTPases, which include RAC, RhoA and CDC42 are both positively and negatively regulated by PH domain-containing GEFs and GAPs that have affinity for PIP_3_ and have been implicated in regulation of LFA-1. RhoA and RAC1 have been implicated in positively regulating LFA-1 avidity by controlling the affinity and clustering of LFA-1 (150, 151). By contrast, CDC42 and RhoH negatively regulate LFA-1 suggesting a complex integrated role of these proteins in LFA-1 regulation (119, 151). TEC kinases regulate cytoskeletal remodelling and LFA-1-mediated adhesion through activation of RHO-family proteins (152–154). In T cells the highest expressed TEC kinases are ITK and RLK, and *Itk* KO cells have decreased adhesion to ICAM-1 (154). RLK does not contain a PH domain, whereas ITK contains a PH domain that binds selectively to PIP_3_ (155), but the role of this in T cell integrin-mediated adhesion is unclear. Similar results have been described for the RHO GEF, VAV1 implicating it in clustering of LFA-1, but it is not known if this effect is PI3K-dependent although VAV1 can be regulated in part by PI3K (156–158). Similarly the RAC-GEF, P-REX1, which also contains a PIP_3_-binding PH domain, has been implicated in LFA-1 affinity and avidity regulation (159). It is likely that TEC kinases, VAV1, and P-REX1 are more important for LFA-1 clustering, and thereby increased avidity, than for affinity regulation as RHO family proteins are known to be important for cytoskeletal remodelling, and recruitment of proteins to the synapse (154). PI3K activity is not sufficient to activate all RHO family proteins (160) and has in some studies been shown to inhibit RAC activity in T cells (128), suggesting a complex interplay of this network of regulators in RHO regulation and downstream regulation of LFA-1.

Intriguingly, DOCK proteins which do not contain PH domains have been suggested to have affinity to PIP_3_ *via* so-called Dock Homology Domains (DHR1) (161). However, the extent of direct PIP_3_ affinity, and whether the affinity is a result of DOCK-proteins interacting with the PH-domain-containing ELMO proteins is still debated (91, 162). DOCK2 was described earlier in the review in the context of chemokine-stimulated LFA-1 activation but does not seem to affect TCR-induced LFA-1 activation, as it seems to be involved in TCR-induced RAC-dependent TCR clustering, without affecting LFA-1 translocation to the IS (91, 163). However, it is possible that this is context-specific, and some subsets thus might be more or less dependent on DOCK2 for efficient LFA-1 activation.

#### Additional PH-Domain Containing Regulators of LFA-1

DNM2 which is known for its role in regulating vesicular traffic, has been suggested to also regulate integrin affinity directly *via* FAK/PYK2- and C3G-mediated RAP1 activation (164). DNM2 has a PH domain, however it does not appear to have affinity for PIP_3_ in screens of PIP_3_-binding (165, 166), and it is therefore unlikely that it is regulated by PI3K.

Interestingly, some RAP GTPase activating proteins (GAPs) have PH domains, including the GAP1-family members RASA3 and RASAL (167–169). In platelets, RASA3 inhibits the affinity of the integrin αIIbβ3 in a PIP_3_-dependent manner (169). How the function of PIP_3_-dependent inhibitors of integrins is coordinated with PH-domain containing proteins that activate integrins is an area of active investigation.

The fact that such a high proportion of LFA-1 regulators contain PH domains suggests key roles for PI3K-mediated signalling in regulating LFA-1, though these may be cell, receptor, and context dependent. Multiple possible mechanisms of PI3K-mediated regulation are plausible; firstly, it is possible that PI3K activity directly activates the PIP_3_-binding LFA-1 regulators by inducing a conformational change in the proteins as is suggested for KINDLIN3 (147). Secondly, PIP_3_ could colocalise proteins that interact and activate each other (As observed during activation of AKT by PDK1). Thirdly, it is possible that microclusters of PIP_3_ colocalise with LFA-1 spatiotemporally during LFA-1 activation. Similarly, it is possible that PIP_3_ inactivates negative regulators of LFA-1 as has been suggested for RASA3-mediated regulation of platelet integrins (169) by similar mechanisms, i.e. conformational inactivation, colocalization of negative regulators with other proteins that inhibit them, or by sequestering the negative regulators from LFA-1 during activation.

## PI3K-Mediated Regulation of Naïve T Cell Migration and Homeostasis

**Figure 5:**
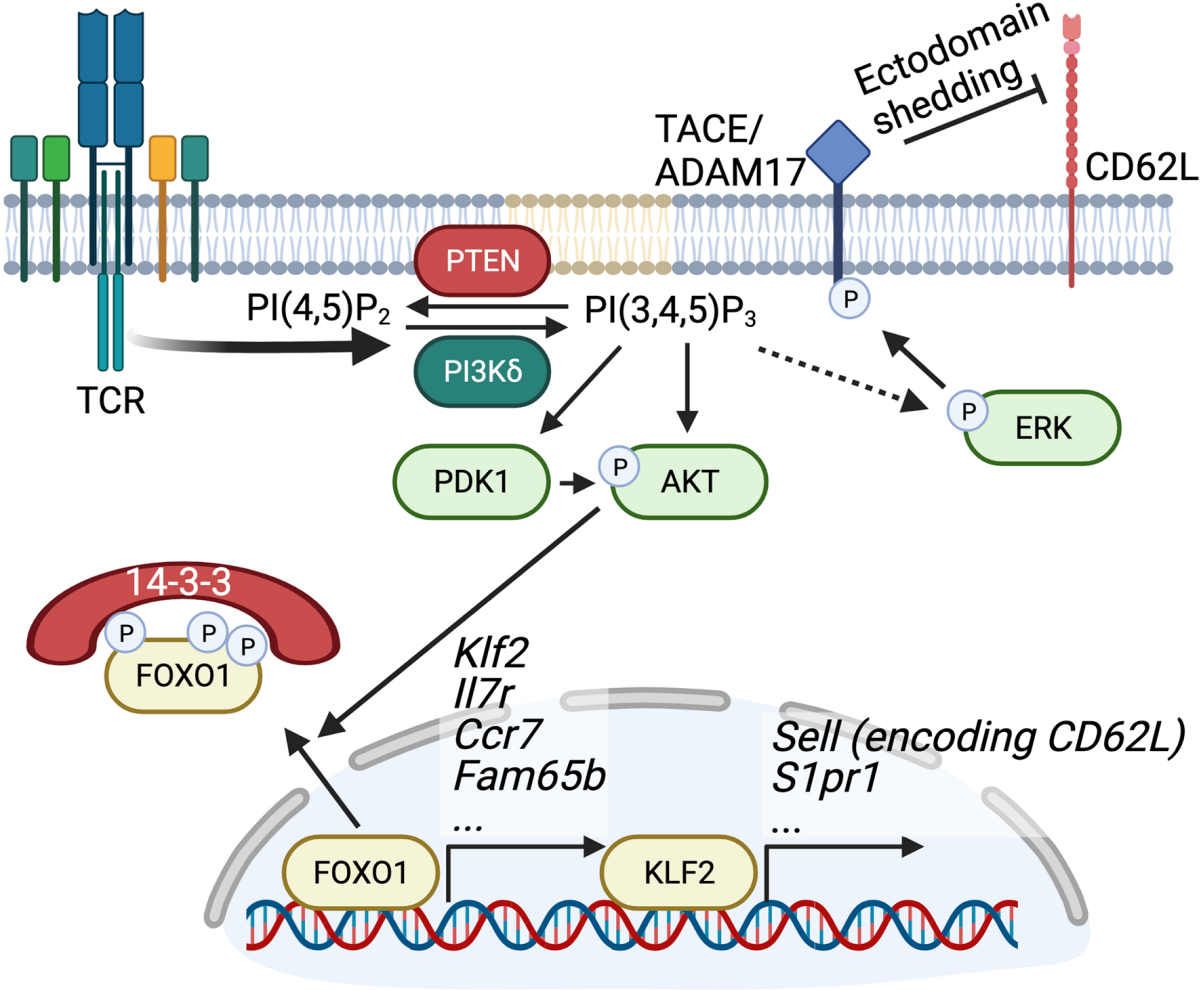
PI3Kδ-mediated regulation of CD62L, CCR7, and S1PR1. PI3K-mediated PIP_3_ production leads to recruitment of PDK1 and AKT, leading to AKT activation. AKT phosphorylates FOXO1, which allows for binding of the 14-3-3 leading to cytosolic sequestration of FOXO1. FOXO1 promotes transcription of *Klf2* (as well as *Il7r*, *Ccr7, Fam65b*). Decreased expression of the transcription factor KLF2 in turn results in decreased transcription of the CD62L encoding gene *Sell* and *S1pr1*. Figure made in BioRender.

The expression of homing molecules CD62L and CCR7 on the surface of naïve T cells is critical for orchestrating naïve T cell trafficking to LNs, where these cells may become activated following antigen encounter and differentiate into effector cells. The maintenance of CD62L and CCR7 expression on naïve T cells is regulated by PI3Kδ signalling and transcription factors of the Forkhead Box protein family, with FOXO1 being a particularly important player. FOXO1 is inhibited by AKT downstream of PI3K (**Figure 5**) (170–172). Once phosphorylated by AKT, FOXO1 is excluded from the nucleus and targeted for degradation (173). Transcriptional activity of FOXO1 is high in naïve T cells and results in robust expression of CD62L and CCR7 through control of KLF2 levels, a transcription factor that drives expression of these key homing molecules (174). In addition, FOXO1 activity is required to maintain IL-7Rα expression, a cytokine receptor key to maintenance of naïve T cell survival and homeostasis (175). Following T cell activation PI3Kδ-mediated signals result in the phosphorylation and inactivation of FOXO1, resulting in a suppression of important FOXO1 and KLF2 target genes involved in regulating migration such as CD62L, CCR7, Fam65b, and others (176–179) (**Figure 5**). As a result of this loss of homing molecule expression, activated T cells are diverted away from entering LNs and instead are biased towards migration into peripheral tissues where they can perform their effector function. In addition to CD62L and CCR7 downregulation, the KLF2 target S1PR1 is also downregulated downstream of PI3Kδ-mediated FOXO1 inhibition (178, 180). Downregulation of S1PR1 expression results in a loss of T cell egress capacity and, combined with negative regulation of CD62L and CCR7 expression, limits the recirculation of activated T cells (**Figure 5**). In fact, suppression of S1PR1 expression is particularly important for the establishment of tissue resident memory T cells (T_RM_), which reside long term at barrier sites and are potent inducers of cell-mediated immunity (181). Therefore, PI3Kδ signals are instrumental in coordinating the acquisition of effector function with necessary changes in cell mobility that are required to execute functional immune responses (**Figure 5**).

Further regulation of CD62L expression on the surface of T cells is mediated through proteolytic cleavage of the ectodomain of CD62L by a process known as CD62L shedding. Following TCR activation, phosphorylation of TNF converting enzyme (TACE)/disintegrin and metalloprotease 17 (ADAM17) drives the trafficking of this protease to the cell surface, facilitating its cleavage of the ectodomain of CD62L (182–184). T cells expressing kinase dead p110δ^D910A^ show impaired shedding of CD62L from the cell surface, suggesting that PI3Kδ activity is critical in this process (185). Mechanistically, it has been shown that phosphorylation of TACE/ADAM17 by mitogen activated protein kinase (MAPK) ERK1/2 is required for the ability of this protease to cleave cell surface molecules like CD62L (186–188) and ERK phosphorylation is impaired in PI3Kδ-deficient T cells (**Figure 5**) (185, 189).

### CCR7 Expression, CD62L-Shedding and LFA-1 Activation – Three Birds, One Stone?

It is intriguing that PI3K signalling regulates multiple processes involved in T cell migration. PI3K-mediated CD62L-shedding and reduced CD62L, S1P1, and CCR7 expression results in decreased LN entry and is an important step in T cell differentiation to effector subsets. Concurrently, as PI3K-signaling increases integrin affinity, PI3K signals can regulate migration and adhesion, including transendothelial migration into LNs. Consequently, inhibition of PI3K or disruption of PI3K signalling will affect all of these rheostats of migration, but not always in predictable ways. Hence, PTEN-deficient T cells with high PIP_3_ levels are excluded from LNs after adoptive transfer (190). Nevertheless, APDS patients suffer from lymphadenopathy and this is revered upon treatment with a PI3Kδ inhibitor (36).

Studies of migration of PI3K-deficient T cells as well as use of inhibitors in mice provide some indication of how inhibition affects T cell distribution *in vivo*. p110γ-deficient T cells show reduced migration towards chemokines, whereas p110δ-deficient T cells respond to chemokines similarly to WT cells (191). Similarly, p110γ selective inhibitors affect responses to chemokines, whereas p110α/β/δ selective inhibitors do not affect responses to chemokines, except for at very high concentrations, likely as a result of off-target effects (192). Following LN entry, p110γ-deficient T cells seem to migrate interstitially similarly to WT T cells, and chemokine-induced interstitial migration seems independent of PI3K signalling (193). However, treatment with Wortmannin as well as disruption of regulatory p85 subunits of class IA PI3K showed that these cells migrated at lower velocities than WT cells, although T cell location within the LN did not seem altered (194). PI3Kδ under steady state does not contribute to T cell migration or chemokine-dependent migration per se, as p110δ^D910A^ T cells migrated like WT T cells in endothelial cell-coated transwell assays as well as following adoptive transfer (195). However, following antigenic challenge, p110δ was required for efficient migration to the site of inflammation and presence of antigen, consistent with a key role for PI3Kδ in regulating integrin affinity (195). Disruption of p110δ results in increased track velocities of OT-II CD4^+^ T cells in LN slices with OVA-pulsed DCs, as a result of decreased interaction times with the peptide-presenting DCs in the slices (128). Similar results have been observed for p110γ-deficient T cells that are defective for antigen-dependent and chemokine-dependent migration of effector CD4^+^ and CD8^+^ T cells (196, 197). Interestingly, CD28 seems to also be important for homing of antigen-stimulated T cells to non-lymphoid tissues, whereas CD28 (Y173F) that is uncoupled from PI3Kδ was defective. This suggests that CD28-mediated activation of PI3K is involved in migration of activated T cells to non-lymphoid sites (198). Consequently, when inhibiting PI3Kδ, homeostatic migration of naïve T cells (T_N_) seems unperturbed (as these have low PI3K activity in the first place), whereas activated T cells show decreased antigen-dependent migration into non-lymphoid tissues.

**Figure 6:**
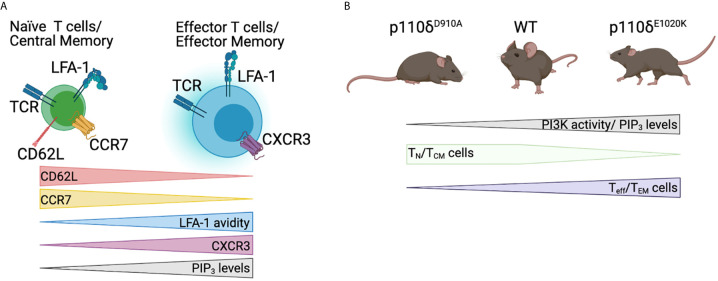
Involvement of PI3K signalling in T cell migration. **(A)** Diagram of relative surface levels of CD62L, CCR7, CXCR3, and avidity of LFA-1 in naïve T cells (T_N_)/Central memory T cells (T_CM_) and effector T cells (T_eff_)/effector memory T cells (T_EM_). **(B)** Spectrum of PI3K activity in PI3K mutant mouse models, and how this affects the levels of T_N_ **/**T_CM_ and T_eff_/T_EM_ cells. Figure made in BioRender.

Central memory T cells (T_CM_) that are CD62L^+^CCR7^+^LFA-1^+^ are consequently supported by PI3Kδ inhibition, whereas effector T cells (T_eff_) and effector memory T cells (T_EM_) (CD62L^-^CCR7^-^LFA-1^+^) are inhibited (**Figure 6A**). This is largely supported by the fact that APDS patients have reduced T_CM_ cells, and increased T_eff_ cells (32), whereas p110δ^D910A^ mice have normal memory T cells, but reduced T_eff_ cells (22) (**Figure 6B**). These PI3K-dependent alterations of T cell memory responses are possibly affected by altered expression of migratory receptors, however, differentiation of p110δ^D910A^ T cells to T_eff_ is largely defective, implicating PI3K more broadly in differentiation and migration.

## PI3K Inhibitors – a Therapeutic Perspective

Because of the important role of PI3K signalling in antigen-dependent migration, PI3Kδ is a promising target for therapies, where broad blockade of T_eff_ and T_EM_ migration is favourable. There is a growing body of evidence that alloreactive memory cells are responsible for allograft rejection Reviewed in (199). Treatments have focused on blocking costimulatory pathways in T cells, but some patients are resistant to these treatments. LFA-1 and/or VLA-4 blocking antibodies (i.e. Efalizumab or Natalizumab) have been used in these patients with some success as these antibodies reduce the migration and activation of memory subsets. However, some anti-LFA-1 or anti-VLA-4-treated patients developed EBV-induced lymphoproliferative disease (200) or the fatal viral brain infection, progressive multifocal leukoencephalopathy (PML) (201), and it has been suggested that targeting TCR-induced inside-out signalling instead of broadly targeting both chemokine, and TCR-induced LFA-1 activation would restrict the blocking to antigen-induced migration of T cells, and therefore potentially limit the risk of PML or EBV-induced lymphoproliferation (202). As PI3Kδ inhibition reduces the antigen-dependent migration of T cells by limiting CD62L and CCR7 downregulation, as well as LFA-1 activity, it is likely that PI3Kδ inhibition would show efficacy in some types of allograft rejection. Indeed, PI3Kδ inhibition decreased chronic rejection of heart allografts in the absence of immunosuppressive treatment by interfering with antigen-dependent migration to the allograft (203). Other groups have also described data supporting the use of PI3Kδ inhibition in treatment of allograft rejection; both in mice with dual PI3K/mTOR inhibition (204) and with p110α/γ (205) or p110δ inhibition alone (206). However, it has been suggested that p110γ deletion is more effective than p110δ deletion, and p110δ deletion and inhibition even seemed to increase allograft rejection (207). Rag KO mice reconstituted with p110δ-deficient CD25^-^ T cells (non-Tregs) prolonged allograft acceptance compared to WTs. This suggests that the negative effect of PI3Kδ inhibition is due to blockade of the immunosuppressive properties of Tregs (207). Further studies will have to evaluate and determine the contribution of CD62L, CCR7 and LFA-1 affinity in allograft rejection, and it will be of interest to systematically determine under what conditions p110δ or p110γ inhibition show efficacy.

Another treatment where T_CM_ cells are favourable to T_eff_ cells, is during adoptive T cell transfer. Studies from Restifo et al. have indicated that adoptively transferred T_CM_ cells are superior to transferred T_eff_ cells both in mice and primates (208–211). The reason why T_CM_ cells elicit a better anti-tumour response is thought to be a result of their circulation to LNs where they persist for longer than short-lived T_eff_ cells (212). Thus, one of the major hallmarks of adoptive cell transfer has been to find ways of differentiating and expanding T cells without terminally differentiating the cells to T_eff_ cells. Multiple ways have been described that support a favourable differentiation profile; Expanding T cells in presence of IL-15, which supports a more central memory-like phenotype, also promotes anti-tumour immunity (213). Similarly, stimulation of WNT signalling (214), inhibition of glycolytic metabolism (215), as well as tethered IL-15 (216), promoted favourable central memory-like phenotypes that augmented anti-tumour immunity. PI3K/AKT inhibition has been shown to favour generation of cells with increased anti-tumour efficacy. AKT inhibition post transfer was shown to promote expansion of favourable T_CM_ cells with improved *in vivo* efficacy (217, 218). Further, PI3Kδ inhibition during expansion of T_H_17 cells followed by inhibition of β-catenin resulted in generation of T_H_17 cells that persisted *in vivo* and elicited heightened anti-tumour immunity (219). Similarly, PI3Kδ inhibition with Idelalisib *ex vivo* before adoptive transfer heightened the anti-tumour response to an even greater extent than AKT inhibition (220). This suggests that PI3K-mediated anti-tumour efficacy in adoptive transfers is partially independent of AKT. Further, the transcription factor TCF7 was increased in the PI3K-inhibited *ex vivo*-expanded T cells, whereas it was not increased to the same extent following AKT inhibition (220). This is surprising as FOXO, which is inhibited by AKT, regulates expression of TCF7. It is possible that additional mechanisms downstream of PI3Kδ are responsible for the increased anti-tumour efficacy seen with PI3Kδ inhibition, and further studies should evaluate the role of other mechanisms in this process, including decreased LFA-1 activation, or the role of other AKT-independent PI3K functions and effectors.

## Summary

In summary we have described how migration is regulated by PI3K signalling in T cells, with a focus on T cell integrin activation. As PI3K activity increases LFA-1 affinity, whilst decreasing CD62L surface levels and CCR7 expression, signalling *via* PI3K is critical in the process of T cell migration following antigen stimulation. We further described how this potentially could be targeted in situations where a naïve/central memory-like phenotype is preferred to effector T cell subsets, such as in allograft rejections and adoptive T cell transfer.

## Author Contributions

KHJ conceived the ideas for the review, outlined, and wrote the manuscript. DPG and JHT contributed with intellectual support, and wrote sections of the manuscript. PLS contributed with intellectual support and suggestions for the manuscript. KO conceived the ideas for the review, wrote sections of the manuscript, and provided intellectual support. All authors contributed to the article and approved the submitted version.

## Funding

This work was supported in part by funding from the intramural programs of NIAID and NHGRI, NIH and from the Wellcome Trust [200925/Z/16/Z].

## Conflict of Interest

KO has received consultancy fees, speaker fees and/or research support from Gilead Pharmaceuticals, Karus Therapeutics and GlaxoSmithKline.

The remaining authors declare that the research was conducted in the absence of any commercial or financial relationships that could be construed as a potential conflict of interest.

## Publisher’s Note

All claims expressed in this article are solely those of the authors and do not necessarily represent those of their affiliated organizations, or those of the publisher, the editors and the reviewers. Any product that may be evaluated in this article, or claim that may be made by its manufacturer, is not guaranteed or endorsed by the publisher.
